# A case of radiation-associated angiosarcoma after breast cancer

**DOI:** 10.1186/s40792-018-0538-9

**Published:** 2018-11-07

**Authors:** Nanae Horisawa, Yayoi Adachi, Masataka Sawaki, Masaya Hattori, Akiyo Yoshimura, Naomi Gondo, Haruru Kotani, Ayumi Kataoka, Kayoko Sugino, Makiko Mori, Mitsuo Terada, Yuri Ozaki, Hiroji Iwata

**Affiliations:** Department of Breast Oncology, Aichi Cancer Center Hospital, 1-1 Kanokoden, chikusa-ku, Nagoya, Aichi 〒464-8681 Japan

**Keywords:** Radiation-associated angiosarcoma, Radiation therapy, Surgery, Local control

## Abstract

**Background:**

Radiation-associated angiosarcoma (RAAS) is a rare subtype of secondary angiosarcoma that is characterized by rapid proliferation and extensive tissue infiltration. Although various treatments for RAAS (such as surgery, chemotherapy, and radiation therapy) have been reported, there is no consensus as to which approach is the best.

**Case presentation:**

A 76-year-old woman presented with right breast cancer (T1N0M0, stage I) 9 years ago. She had undergone breast-conserving surgery and sentinel lymph node biopsy and was receiving adjuvant chemotherapy and radiation therapy for the malignancy. Six years after presenting with the tumor, she developed pigmented skin and was diagnosed with a RAAS; this angiosarcoma recurred three times within 2 years. The angiosarcoma was resected each of the three times, after which adjuvant radiation therapy was performed. At 76 years old, the patient developed a new mass on her chest skin in the vicinity of the scar. Angiosarcoma was diagnosed following a pathology report, which resulted in a second diagnosis of recurrent RAAS again since the diagnostic criteria were met. After extensive resection of the irradiated area, the patient has remained free of angiosarcoma for the last 3 years.

**Conclusion:**

Resection of the entire irradiated field is critical for successful treatment of RAAS.

## Background

Angiosarcomas are rare malignancies that arise from the endothelial cells that line vascular channels. These tumors are characterized by their rapid proliferation and extensive infiltrating growth. Approximately 8% of all angiosarcomas arise in the breast [1, 2].

Angiosarcomas are either primary tumors of unknown etiology or secondary tumors that arise via several routes. There are two subtypes of secondary angiosarcoma. The first is known as Stewart-Treves syndrome and arises in the lymphedematous upper extremity following mastectomy and irradiation [1, 3]. The second type is the radiation-associated angiosarcoma (RAAS), which arises in tissue at the site of a prior irradiation treatment. Several studies have reported that radiation therapy is a risk of angiosarcomas [4].

Several investigators have pointed out an increased incidence of RAAS after treatment for breast cancer. Huang et al. found that women treated for breast cancer had higher incidence of soft tissue sarcoma and angiosarcoma [5]. A recent review of Surveillance, Epidemiology, and End Results (SEER) data demonstrated a ninefold increased relative risk of RAAS of the breast and chest wall following irradiation of the primary breast cancer [4].

Although treatments for RAAS have been controversial, surgery remains the current standard of care. While mastectomy is reportedly more effective than tumorectomy, it is not in itself sufficient to treat RAAS [6]. Defining the optimal treatment strategy for RAAS is also challenging due to the rarity of this tumor type [7]. We herein report a case of repeated local recurrence of RAAS that was successfully treated by surgery.

## Case presentation

**Fig. 1:**
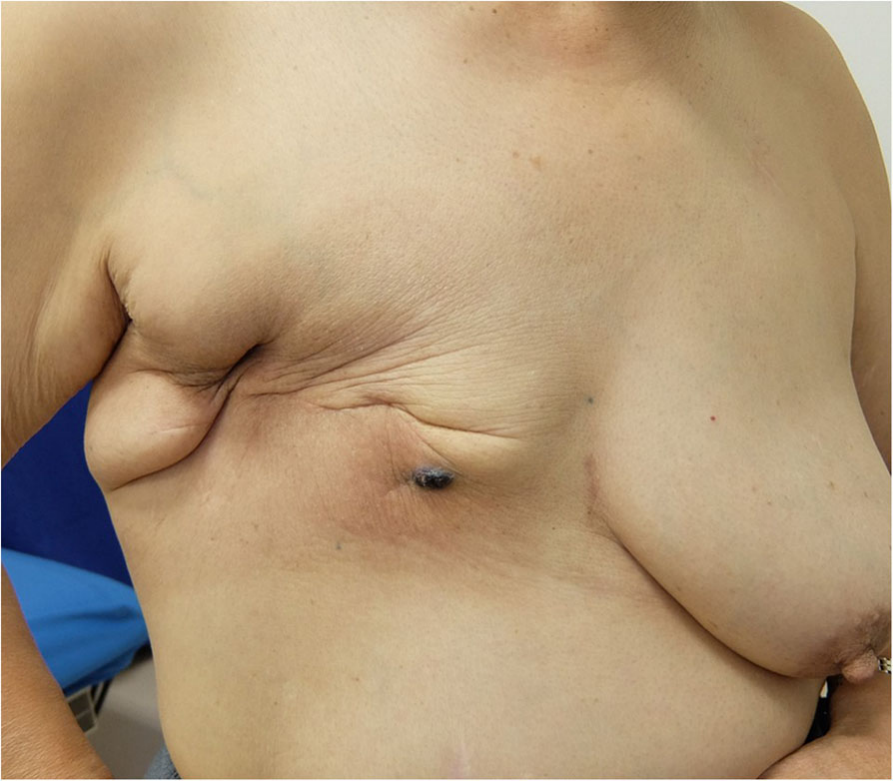
Light pigmentation and a dark red tumor localized to the right chest wall of the patient

**Fig. 2:**
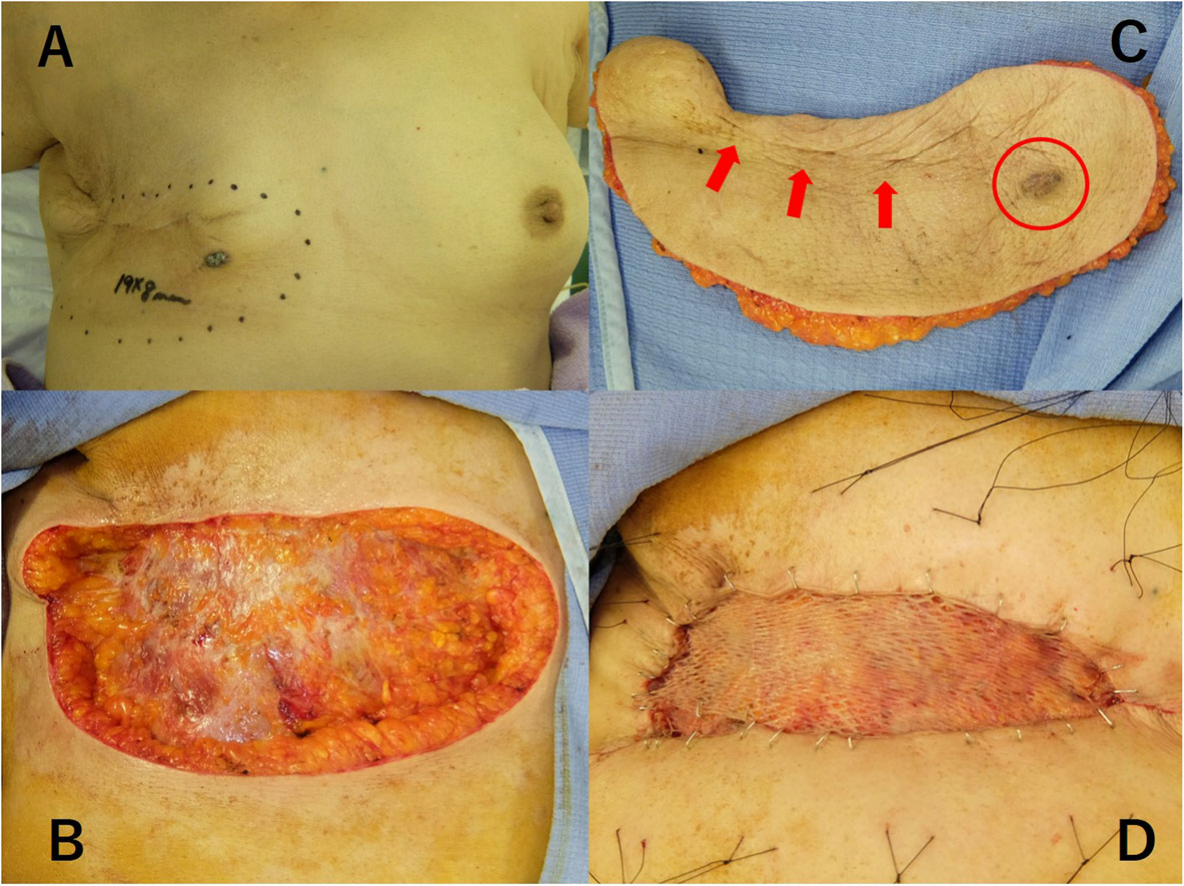
**a** Before the operation. The mark includes the irradiated area. **b** Resected specimen (irradiated area including tumor). The tumor is shown in red circle, and the scar is indicated by red arrows. **c** The irradiated skin of the chest wall was resected as much as possible. **d** Epithelialization. We conducted split-thickness skin graft using collected skin from the patient’s right thigh

**Fig. 3:**
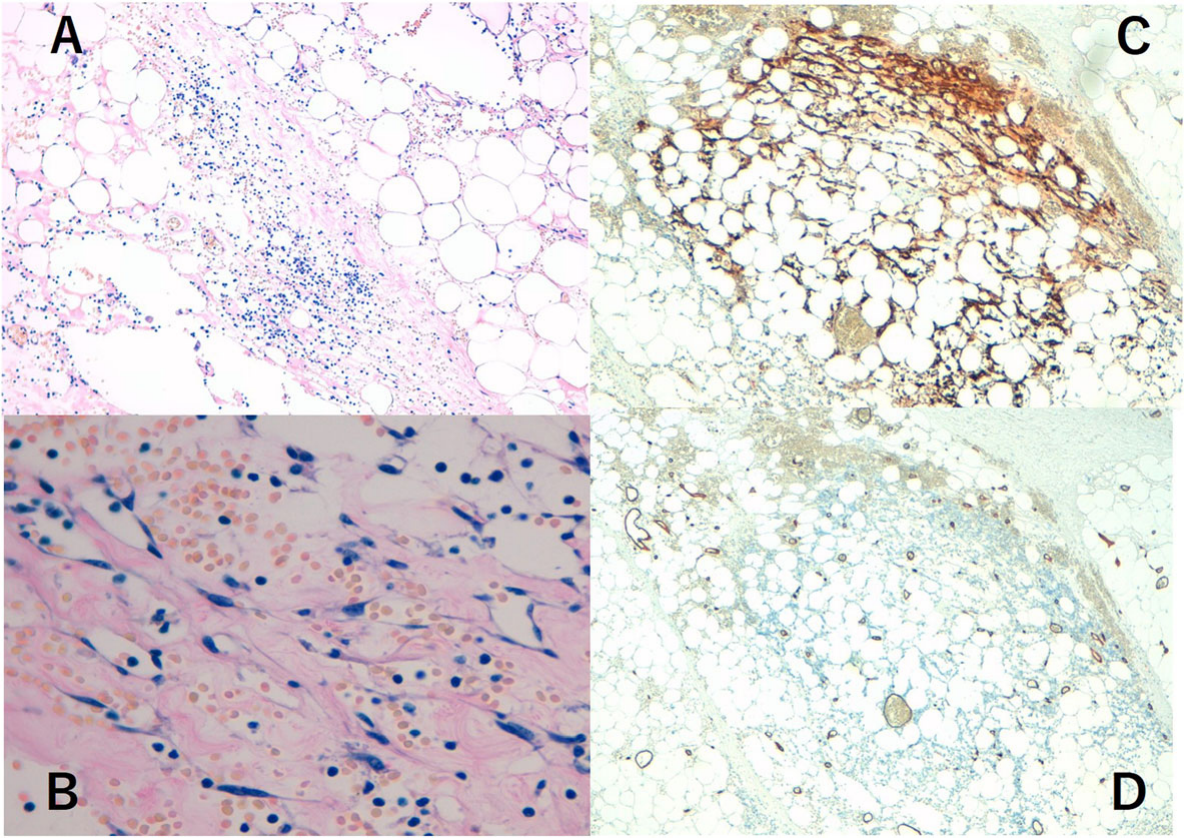
**a** Hematoxylin and eosin staining. There were many spindle cells and dilated vascular channels. **b** Magnification of hematoxylin and eosin staining. Immunostaining of the tumor was CD31-positive (**c**) and mildly positive for CD34 (**d**)

**Fig. 4:**
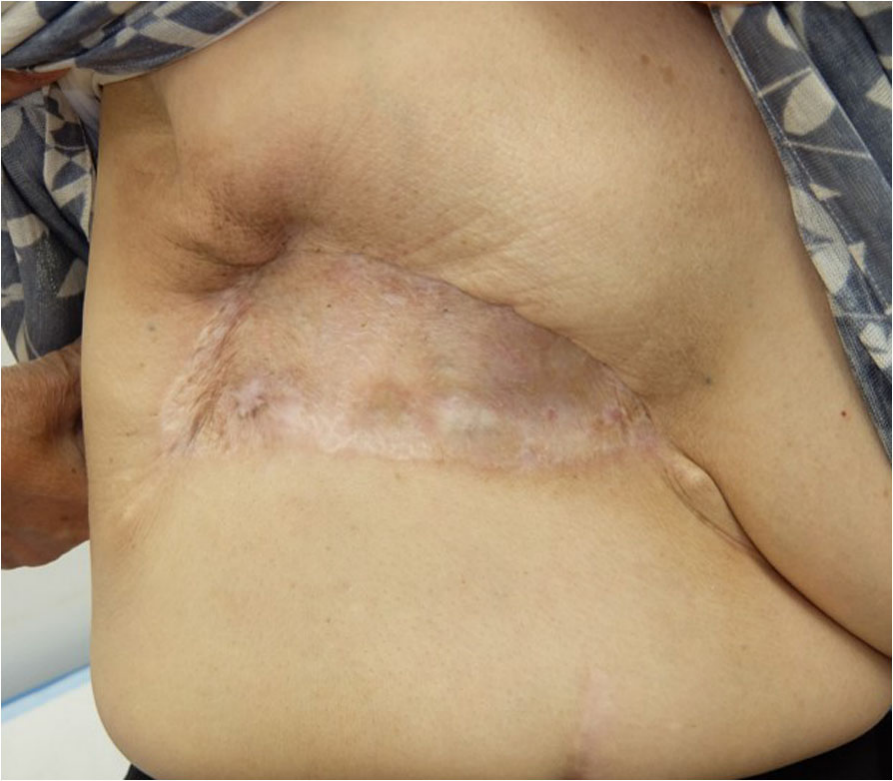
After the operation. The patient has not suffered an RAAS relapse in the last 3 years

A 76-year-old woman presented to our hospital with a mass occurring on the skin of her right chest wall. She had been diagnosed with right breast cancer (T1N0M0, stage I) 9 years previously and had received breast-conserving surgery, sentinel lymph node biopsy, and adjuvant chemotherapy and radiation therapy for the residual whole right breast at a previous hospital. She then developed pigmented skin on her right breast 6 years after surgery, and this lesion was diagnosed as an angiosarcoma. The patient underwent a breast mastectomy to treat for RAAS. Following this, however, the angiosarcoma on her chest wall recurred three times within 2 years. The angiosarcoma was resected each time, and she received radiation therapy to her chest wall after the third operation. Four years after the first occurrence of RAAS, we observed light pigmentation and a dark red tumor (gross diameter of 5 mm) on her right chest wall (Fig. 1). Clinically, recurrence of RAAS was suspected, and recurrence of angiosarcoma was diagnosed by biopsy. We considered that it was necessary to remove the irradiated skin as much as possible in order to cure the RAAS. After extensive resection of the irradiated skin and tumor, new skin collected from her right thigh was grafted to the site (Fig. 2). Pathologically, the tumor size was 6 mm and the surgical margin was negative. Histologically, there were many spindle cells and dilated vascular channels. Immunostaining showed that the tumor was CD31-positive and mildly positive for CD34 (Fig. 3). Ki-67 index was also high. It was revealed that there is no inconsistency as recurrence of RAAS is pathological. After the operation, the patient was hospitalized for 30 days and did not experience any complications. Although some reports suggest chemotherapy can be used to treat RAAS, we considered that this option would offer little benefit in this case, because the patient was elderly and had a history of cerebral infarction. Indeed, the patient has remained angiosarcoma-free for the last 3 years following our intervention, even without chemotherapy (Fig. 4).

## Discussion

RAAS is a rare type of secondary angiosarcoma with an incidence that varies from 0.05 to 1.11% [1, 8]; the malignancy is often seen in elderly patients [9]. In 1984, Cahan et al. defined RAAS as a sarcoma that arises in an irradiated field of tissue. They also defined RAAS as pathologically confirmed breast or chest wall angiosarcoma arising within a previously irradiated field, in patients with a history of radiation therapy and a latency period of at least 3–4 years from their last radiation therapy [10, 11]. Because the current case met all the above criteria, we diagnosed it as a clinical recurrence of RAAS.

The latency for RAAS occurrence in the irradiated area is 10–12.5 years after the first radiation therapy [9, 12, 13]. Pathologically, angiosarcoma presents as solid sheets of anaplastic endothelial cells that form complex vascular channels. Vascular endothelial markers, for example, CD31, CD34, VIII-related antigen, and *Ulex* lectin stains, are useful for diagnosis [12]. Sometimes D2-40-reactive lymphatic endothelial markers are seen.

RAAS is associated with high mortality. For example, Colwick et al. reported a 5-year survival rate of 15–20% [12]. Gervais et al. reported that a 5-year OS is 44% and DFS is 23% [6], Torres et al. reported a 10-year OS of 54% [13], and Depla et al. reported a 5-year OS of 43% [7].

RAAS is usually sporadic and spread across multiple sites in the irradiated field and has a high local recurrence rate. With regard to the pattern of the first recurrence of angiosarcoma (including RAAS and sporadic angiosarcoma), Gervais et al. reported that the local recurrence rate was higher than the distant recurrence rate (46% vs. 13%) [6]. Torres et al. reported that local recurrence following R0/R1 resection was observed in 48% of patients compared with 27% of distant metastasis, after a median follow-up of 10.8 (range, 2.4–31.8) years. Additionally, 24% of the patients developed second local recurrences [13]. The authors suggested that this is because microsatellite lesions spread beyond the apparent R0 margins in RAAS in the irradiated area. Ketja et al. also reported the local recurrence rate after R0 resection was 54% [14]. According to their study, the median survival rate in patients who did not develop a local recurrence after R0 resection was higher than those of patients who developed a local recurrence (40.5 months vs. 20 months). From these studies, it is clear that RAAS has a high local recurrence rate even if the surgical margin is negative, and prevention of local recurrence is vital in order to prolong OS.

Although many treatment modalities for RAAS have been proposed, there is no consensus for the optimal treatment because clinical cases are very rare, and this precludes rigorous statistical analysis. Total mastectomy could be considered the current standard treatment for RAAS following breast conservation. However, mastectomy alone is still associated with a high risk of local recurrence; in one report, 73% of patients undergoing mastectomy alone developed further RAAS [1]. In our case, the patient had repeated local recurrence of RAAS, despite the fact that she underwent a mastectomy for her first occurrence of RAAS. These data show that the local control of RAAS is very difficult.

Li et al. reported on surgery-specific risk stratification in the context of RAAS. In this retrospective study, 76 women with RAAS were enrolled and randomized into two groups, the “radical group” and a “conservative group.” The “radical” resection was defined as resection of all the previously irradiated area plus mastectomy. Consequently, the “conservative group” was defined as standard resection such as mastectomy or tumorectomy without total resection of the irradiated area. The radical surgery group had a lower 5-year cumulative local recurrence incidence compared to the conservative surgery group (23% vs. 76%, *P* < 0.001) and distant metastasis incidence (18% vs. 47%, *P* = 0.02). Five-year disease-specific survival (DSS) for the radical group was much higher than that for the conservative group (86% vs. 46%, *P* < 0.001). In addition, patients who underwent conservative surgery with negative margins had a worse DSS than those in the radical group [15]. This study suggests that an extended resection of irradiated area is an effective treatment for RAAS.

Chemotherapy has also been used to treat RAAS. Fujisawa et al. reported that chemoradiotherapy with taxane is superior to conventional surgery and radiation therapy for angiosarcoma. In this retrospective study, the response rate of chemotherapy was 94%, and a 5-year OS for the chemotherapy group was statistically higher than that for the surgery group (56% and 8%, respectively; *P* < 0.01). However, this study included not only RAAS but also primary angiosarcoma [16]. In contrast, Colwick et al. found that chemotherapy has a minimal benefit (only 17–34% response rates) [12]. Therefore, the true benefit of chemotherapy for RAAS treatment is uncertain. Torres et al. reported on the impact of adjuvant chemotherapy for RAAS and found that it reduces local recurrence rate; however, they concluded that adjuvant chemotherapy was not significantly associated with a better prognosis [13]. Additionally, the optimal chemotherapy regimen is also unclear [2]. Moreover, angiosarcomas overexpress vascular endothelial growth factor (VEGF). Agulnik et al. reported the efficacy of bevacizumab for the treatment of advanced angiosarcoma. They enrolled 32 patients with advanced angiosarcoma which could not be resected surgically, prospectively. The result was that 17% of patients had a partial response and 50% of patients showed stable disease with a mean time to progression of 26 weeks. They concluded that bevacizumab was effective for advanced angiosarcoma [17]. On the contrary, Ray-Coquard et al. reported on a study comparing paclitaxel with or without bevacizumab regimen to treat for advanced angiosarcoma including RAAS. Six-month progression-free survival rate was 54% in the paclitaxel alone group and 57% in the paclitaxel with bevacizumab group. This indicates that paclitaxel with bevacizumab regimen is not superior to paclitaxel alone [18]. Although both studies were prospective and including not only primary angiosarcoma and but also RAAS, it appears that more data is needed to reveal the effectiveness of bevacizumab to treat RAAS.

Although several reports recommend surgery and adjuvant radiation therapy with large doses, no formal radiation therapy trials have been done [19]. Moreover, repeated exposure of the area to radiation is likely to result in local recurrences [12]. Therefore, we consider that the use of radiation therapy for RAAS is also controversial. Additionally, the relationship between RAAS and radiation dose remains unclear. In our case, the patient had been subjected to radiation therapy twice, and RAAS had clearly arisen in the irradiated area of her chest wall. This indicates that the use of radiation therapy to treat RAAS may increase the risk of local recurrence or another new occurrence of RAAS.

In summary, RAAS has a high local recurrence rate even if the surgical margin was negative, because it spreads throughout the irradiated area. In the current case, the patient had repeated local recurrences of RAAS that arose in the irradiated area on three successive occasions. Therefore, we suggest resecting as much as possible of the irradiated area as the most effective treatment for RAAS. Although this patient could not be treated with adjuvant chemotherapy due to her age and history of cerebral infarction, she was completely cured as a result of the “extensive” surgery. We conclude that the resection of the entire irradiated area is the most effective treatment for control of local recurrence and extension of survival time in RAAS patients.

## Conclusion

Here, we reported a rare case of repeated local recurrence of RAAS that was controllable by surgery. We conclude that removal of the entire irradiated field is the most important treatment for this malignancy. Further studies of additional RAAS cases will be required in order to define the optimal treatment regimen.

## Abbreviations

DFS: Disease-free survival
DSS: Disease-specific survival
OS: Overall survival
PFS: Progression-free survival
RAAS: Radiation-induced angiosarcoma
